# The source attribution analysis revealed the prevalent role of poultry over cattle and wild birds in human campylobacteriosis cases in the Baltic States

**DOI:** 10.1371/journal.pone.0235841

**Published:** 2020-07-09

**Authors:** Mihkel Mäesaar, Triin Tedersoo, Kadrin Meremäe, Mati Roasto

**Affiliations:** 1 Chair of Food Hygiene and Veterinary Public Health, Institute of Veterinary Medicine and Animal Sciences, Estonian University of Life Sciences, Tartu, Estonia; 2 Veterinary and Food Laboratory, Tartu, Estonia; United States Department of Agriculture, Agricultural Research Service, UNITED STATES

## Abstract

The reservoir and source of human campylobacteriosis is primarily considered to be poultry, but also other such as ruminants, pets and environmental sources are related with infection burden. Multilocus sequence typing is often used for *Campylobacter* epidemiological studies to determine potential sources of human infections. The collection of 420 *Campylobacter jejuni* isolates with assigned MLST genotype from poultry (n = 139), cattle (n = 48) and wild birds (n = 101) were used in source attribution analysis. Asymmetric island model with accurate and congruent self-attribution results, was used to determine potential sources of human *C*. *jejuni* infections (n = 132) in Baltic States. Source attribution analysis revealed that poultry (88.3%) is the main source of *C*. *jejuni* human infections followed by cattle and wild bird with 9.4% and 2.3%, respectively. Our findings demonstrated that clinical cases of *C*. *jejuni* infections in Baltic countries are mainly linked to poultry, but also to cattle and wild bird sources.

## Introduction

Campylobacteriosis is the most commonly reported zoonosis in the European Union with 246,571 confirmed human cases, which represents a notification rate of 64.1 per 100,000 population in 2018 [1]. *Campylobacter jejuni* was reported in 83.9% of the confirmed cases where species information was provided (55.2%). In the Baltic states there are approximately 6 million inhabitants among whom 1,417 cases were confirmed in 2018 with an average notification rate of 22.8 per 100,000 population. All Estonian (n = 411), 97,8% of Latvian (n = 87) and 99.4% of Lithuanian (n = 919) cases were confirmed with notification rates 31.2, 4.5, 32.7 per 100,000 population, respectively. In 2018 Lithuania reported five *Campylobacter* related food-borne outbreaks (FBO) with 10 human cases in combined. At the same time no *Campylobacter* related FBOs were reported in Estonia nor Latvia [1]. *Campylobacter* related infections are at high clinical importance as accompanying symptoms vary from mild fever, abdominal pain, vomiting, dehydration to bloody diarrhea and in some cases sever neurological disorder Guillain-Barré syndrome as post-infectious complication [2,3]. Therefore, the determining the source of human *C*. *jejuni* infection has high public health importance.

Multilocus sequence typing (MLST) data has been successfully used for attribution studies to determine potential links between the sources and clinical isolates [4]. Several overview studies have been recently published regarding epidemiology and MLST genotype diversity of *C*. *jejuni* in the Baltic countries by Mäesaar et al [5], Meistere et al [6] and Aksomaitiene et al [7]. Although the studies demonstrated overlap between *C*. *jejuni* MLST genotypes isolated from human patients and genotypes found in poultry and cattle, there has not been conducted source attribution analyses of clinical Baltic *C*. *jejuni* isolates.

The aim of this study was to comprehensively describe the entire Baltic region’s *C*. *jejuni* MLST genotype diversity within poultry, cattle and wild bird sources by aggregating data from surveys conducted in Estonia, Latvia and Lithuania. To our knowledge this is the first time when population genetic analyses was conducted to attribute clinical Baltic *C*. *jejuni* isolates to their most likely sources.

## Methods

### *C*. *jejuni* isolates

The collection of *C*. *jejuni* isolates (n = 420) with assigned MLST genotype from human patients (n = 132), poultry (n = 139), cattle (n = 48) and wild birds (n = 101) were obtained from three previously published studies [5–7] (S1 File). Sources with less than 40 strains were discarded. Isolates from outside the Baltic region was not included as geographical distance has potential to cause bias in attribution analyses [4].

### Population diversity analysis

Full minimum spanning tree (MST) of MLST allele differences was constructed using goeBURST algorithm [8] as implemented in PHYLOViZ v2.0 [9] (S4–S6 Files).

### Source attribution modelling

To attribute human *C*. *jejuni* cases to potential sources the evolutionary asymmetric island (AI) model, which accounts mutation, recombination and migration rates, was used as implemented in software iSource (downloaded from the website http://www.dainelwilson.me.uk/software.html) [10]. The model was used with following parameters: 1,000,000 iterations, recording state of the Markov chain Monte Carlo (MCMC) every 50 iterations and utilizing a symmetric Dirichlet prior with parameter 1 (all sources are considered equally likely) [10]. Convergence of the model was assessed using 10 different runs (S7 and S8 Files). Self-attribution tests were performed as described by Berthenet et al [2] using the 288 isolates from three potential sources (poultry, cattle, wild birds). For the self-attribution analyses three different initial datasets were created. Each dataset consisted 20 randomly selected isolates from each of the three mentioned reservoirs. Randomly selected isolates with known sources were assigned as unknown samples in iSource input file. The model was used with the previously described parameters in 10 separate runs for each three datasets to assess model convergence (S7 and S9–S11 Files). Isolates were attributed to the corresponding source when assigned posterior probability of one potential source was higher than other two [10].

All the results were calculated from iSource output files using R Statistical Software v3.6.2 [11] with modified script provided with the iSource software (S12 File).

### Statistical analyses

The Czekanowski proportional similarity index (PSI) was calculated using Excel (Microsoft Corporation, Washington, USA) to compare distribution of STs from different sources. PSI ranges from 1 for identical ST distribution to 0 for distribution with no common genotypes [12].

Genetic divergence between sources was further analyzed with analysis of molecular variance (AMOVA) [13] as implemented in GenAlEx v6.5 [14] using haploid alleles. Significance was assessed by permutation test, using 999 permutations. Pairwise significant differentiation between sources was calculated and the significance of PhiPT (an analogue of FST) values was assessed as previously described (S2 and S3 Files).

## Results

### Population diversity analysis

**Fig 1:**
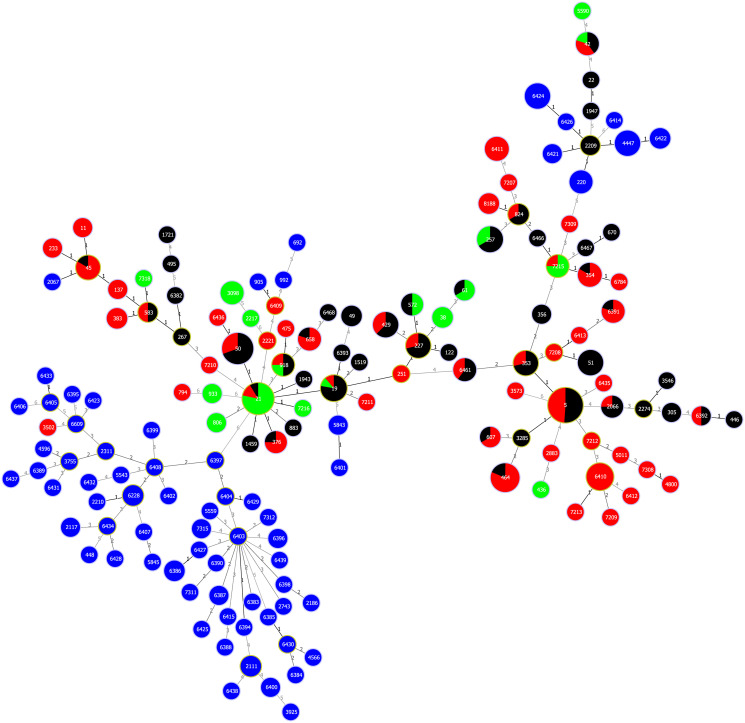
Overlap between human, poultry and cattle isolates. goeBURST full Minimum Spanning Tree of 420 Baltic *C*. *jejuni* sequence types (ST) allelic profiles. Nodes are named after STs and colour-coded according to isolate sources: poultry (red); cattle (green); wild bird (blue), clinical isolates (black). Links are labeled with number of allelic differences. Data associated with the figure are presented in the S1 and S4–S6 Files.

The most prevalent CC among 28 assigned complexes in the dataset (n = 420) was ST-21CC (n = 70; 16.7%) followed by ST-353CC (n = 64; 15.2%) and ST-179CC (n = 26; 6.2%). CCs were not assigned for the 99 isolates from human (n = 4), poultry (n = 27), cattle (n = 8) and wild bird (n = 60) sources. From the 158 assigned STs, the most common was ST-5 (n = 45; 10.7%), followed equally by ST-21 (n = 23; 5.5%) and ST-50 (n = 23; 5.5%). Frequency of STs was skewed as 34.8% genotypes covered 74.5% of the dataset. The overlap between human and poultry and/or cattle isolates were substantial as 25 genotypes covered more than half of the combined dataset (Fig 1).

Clinical *C*. *jejuni* isolates from human (n = 132) were assigned to 49 different STs. The most prevalent STs were ST-5 (n = 23; 17.4%) followed by ST-50 (n = 16; 12.1%) and ST-51 (n = 8; 6.1%). Poultry isolates (n = 139) consisted 53 different MLST genotypes. The most common genotypes in the sample were ST-5 (n = 22; 15.8%), ST-464 (n = 13; 9.4%) and ST-6410 (n = 11; 7.9%). Cattle isolates (n = 48) divided into 17 STs with most prevalent being ST-21 (n = 18; 37.5%) followed by ST-3098 (n = 5; 10.4%) other STs had less than 5 isolates assigned to them. Wild bird isolates (n = 101) had most diverse set of STs consisting 68 different STs. Top three assigned STs were ST-4447 (n = 8; 7.9%), ST-6424 (n = 8; 7.9%) and ST-220 (n = 5; 5.0%). There were 103 singleton STs consisting only one isolate per genotype in the combined dataset. Per source the singleton STs were distributed as follows human (n = 29; 22.0%), poultry (n = 31; 22.3%), cattle (n = 8; 16.7%) and wild birds (n = 54; 53.5%).

Pairwise PSI indexes calculated for different sources ranged from 0 to 0.41. Highest similarity was detected between human and poultry isolates (PSI = 0.41; 21 shared STs) followed by human and cattle (PSI = 0.14; 7 shared STs) and poultry and cattle (PSI = 0.06; 5 shared STs). Wild bird isolates were dissimilar from all the other sources (PSI = 0). Among human, poultry and cattle there were four overlapping STs (ST-19; ST-21; ST-42; ST-918) with total of 41 in combined (9.8%).

More than half of the assigned genotypes (n = 133) were host restricted. The most common genotype associated only with human isolates was ST-51 (n = 9; 18.4%), altogether there were 25 genotypes (n = 38; 77.6%) restricted to human source. ST-6410 (n = 11; 7.9%) was most prevalent genotype out of 31 (n = 53) associated to poultry source. Nine (n = 17) out of 17 genotypes was restricted to cattle, with ST-3098 (n = 5; 10.4%) being the most common. None of the 68 wild bird genotypes overlapped with STs detected from other sources. Nevertheless, some clinical isolates clustered closely with wild bird isolates (Fig 1). For example, clinical *C*. *jejuni* isolates assigned to ST-2209 had only one allelic difference between four wild bird associated STs (ST-220; ST-4447; ST-6421; ST-6426).

### Source attribution analysis

AMOVA showed that 12.5% (p = 0.001) of the molecular variation originated among the sources. Pairwise PhiPT values for all sources ranged from 0.019 to 0.197 and were statistically significant (p = 0.001). Lowest variation 1.9% were between human and poultry followed by 9.7% for human and cattle and 13.1% between poultry and cattle sources. Molecular variation between wild bird and poultry, human and cattle sources were 16.4%, 18.3% and 19.7%, respectively.

**Fig 2:**
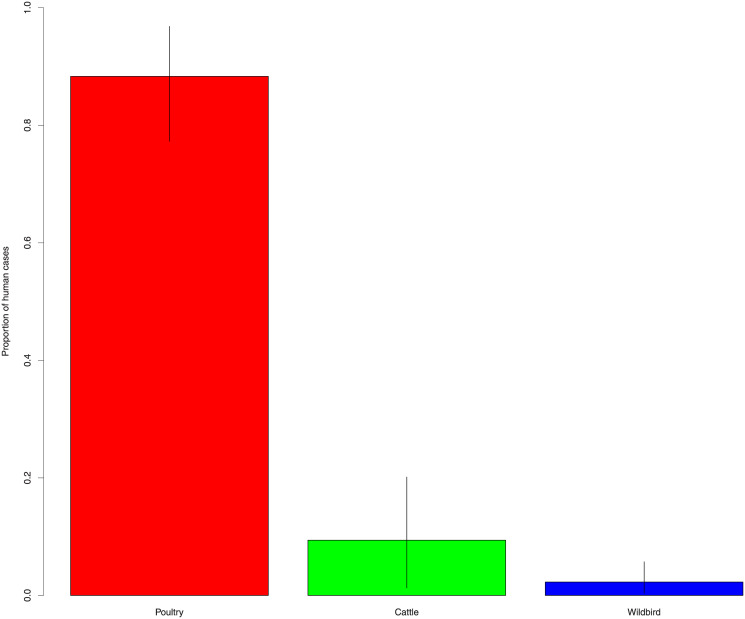
Clinical *C*. *jejuni* cases attributed to poultry, cattle and wild bird. Estimated proportion of human cases with 95% credible intervals for each attributed source: poultry (red), cattle (green) and wild bird (blue).

**Fig 3:**
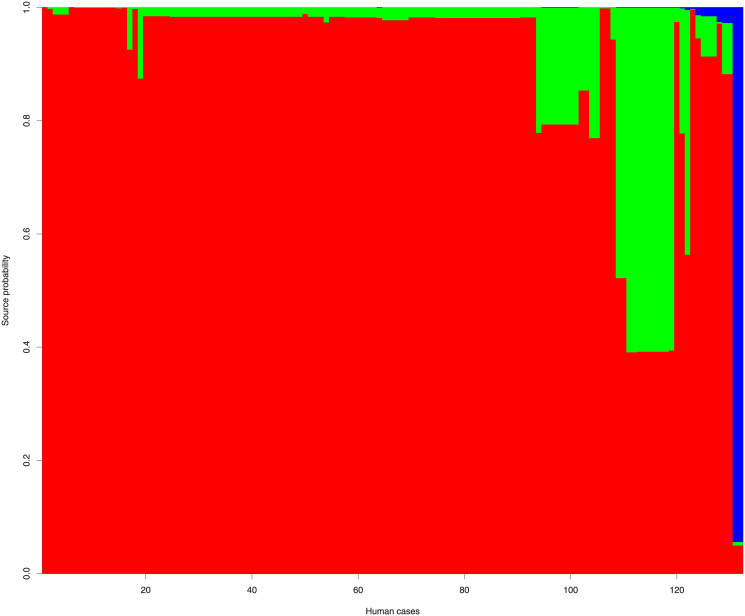
The source probabilities of human *C*. *jejuni* cases. 132 human cases (vertical columns ordered horizontally for better visualization). Colour-coded according to isolate sources: poultry (red); cattle (green); wild bird (blue).

According to source attribution analysis the proportion of clinical *C*. *jejuni* cases attributed to poultry, cattle and wild bird were 88.3% (95%CI: 77.3–96.8), 9.4% (95%CI: 1.3–20.1) and 2.3 (95%CI: 0.4–5.7), respectively (Fig 2). Two out of 21 Latvian clinical *C*. *jejuni* isolates were attributed to cattle and 19 to poultry source. Eight out of 10 Estonian *C*. *jejuni* isolates originated from human patients were attributed to poultry and two to cattle source. Two out of 101 Lithuanian clinical isolates attributed to wild bird, 94 to poultry and five to cattle (Fig 3).

Self-attribution analyses results showed good congruence with >90% correct self-assignments for isolates from different sources.

## Discussion

Probabilistic AI attribution model used in present source attribution study has potential to broaden our knowledge regarding epidemiology and relative contribution of probable reservoirs of *C*. *jejuni* human infections in the Baltic States. Regardless of the above, like other probabilistic attribution models AI model has certain limitations and assumptions, which are important to consider in the context of the results. First, source reservoirs are predefined and consequently excluding unknown sources [2]. The *Campylobacter* research conducted in the Baltic States has mainly been focused to human, poultry, cattle and wild bird sources [5–7]. There is very limited or no information regarding *C*. *jejuni* in other possible *Campylobacter* sources such as pigs, pets and specific environmental sources (natural water, sewage sludge). Consequently, we focused on poultry, cattle and wild birds as potential sources of human *Campylobacter* infections. Second, the underlying directionality assumption in present study considers humans as the recipients of *Campylobacter* infections, but this may not always be the case. For example, there is possibility for humans to infect animals and wild birds through watering food crops using irrigation water contaminated with human waste [15]. Third, generalist strains common in all hosts could be difficult to assigned to certain source with high probability [2]. This problem did not occur in present study.

Our results show that genotypic population diversity of *C*. *jejuni* originating from the Baltic States is diverse to a considerable extent, nevertheless calculated PSI values demonstrated an overlap between isolates from different sources. It is important to emphasize that wild bird source consisted wide range of singleton STs that were not assigned to any known CCs. Hughes et al [16] has suggested that possible emergence of new unique wild bird related STs could be explained with recombination occurring during coinfection with more than one strain. Nevertheless, pairwise PhiPT values obtained from AMOVA that were consistent with previously mentioned index values. AMOVA results indicated that there was significant difference between different sources, although pairwise PhiPT values were in a wide range all the values were statistically significant. Above-mentioned is one of the main prerequisite to conduct source attribution analysis of clinical cases with some degree of precision [10].

Self-attribution was conducted to validate the source attribution results as suggested by Cody et al [4]. As self-attributed strains from poultry, cattle and wild birds were correctly assigned to known sources with more than 90% accuracy, therefore the bias was not corrected as the risk of under-estimation of attribution to different sources was not substantial [2].

Our source attribution analysis results showed that poultry is the main source of *C*. *jejuni* infection in the Baltic States, later is supported with several other studies applying AI model [10,17–25]. Cattle was the second important source of human infections followed by wild bird associated clinical *C*. *jejuni* strains. Similar top three sources pattern was observed in multiple other studies, although some of them combined cattle and sheep as ruminant source and wild bird together with environmental samples as environmental source, while other did not concatenate latter mentioned sources [19–21,24,25].

Previous studies performed in Estonia [5] and Lithuania [35] showed highest similarity between human and poultry isolates with overlapping STs and antimicrobial profiles. According to our source attribution analysis the proportion of clinical cases attributed to poultry was 88.3% (95%CI: 77.3–96.8) almost identical results have been observed by two other studies with similar source classifications using AI model. French et al [19] attributed 75.0% (95%CI: 64.4–85.4) and Sheppard et al [24] 78.0% cases to poultry isolates. Mullner et al [22] used modified Hald model that uses Bayesian approach which assigned 80.0% *C*. *jejuni* strains to poultry source. All three of the above-mentioned results remain within the 95% confidence interval. Three other studies conducted by Bessell et al [26], Kovac et al [27] and Lévesque et al [28] used similar sources but with STRUCTURE algorithm and assigned 46.4%, 58.0% and 64.5% clinical *C*. *jejuni* cases to poultry reservoir, respectively. Sheppard et al [24] demonstrated that AI algorithm assigns more cases to poultry source than STRUCTURE, while having greater self-attribution accuracy than STRUCTURE algorithm for *C*. *jejuni*.

Clinical cases of *C*. *jejuni* were attributed to cattle source in 9.4% (95%CI: 1.3–20.1) of cases. Sheppard et al [24] and French et al [19] observed similar results 17.0% and 18%, respectively. The latter result applies to sheep and cattle. Modified Hald model applied by Mullner et al [22] attributed 10.0% of cases to cattle, while STRUCTURE algorithm used by Bessel et al [26], Kovac et al [27] and Lévesque et al [28] showed higher proportion assigned to the cattle source 31.0% (cattle and sheep combined to ruminant source), 34.8% and 25.8%, respectively for the reasons mentioned above.

Wild bird source was assigned to human cases in 2.3 (95%CI: 0.4–5.7) of cases. Five out of six studies got results that were within 95% confidence interval of our finding 1.9% [26], 2.0% [19], 2.3% [28], 1.0% (environmental source) [22] and 4.0% (wild bird and environmental source combined [24]. Kovac et al [27] combined water and wild bird sources, therefore reporting higher (7.2%) result.

In view of the above our source attribution analysis results are consistent with previously conducted studies.

Poultry and cattle are known reservoirs [29,30] for *C*. *jejuni*. Wild bird especially shedding of wild bird faeces is important reservoir of *C*. *jejuni* infections [15,31]. Whiley et al [32] describes wild bird faeces at playgrounds as emerging environmental source of campylobacteriosis, which is also supported by Abdollahpour et al [33]. Especially vulnerable are children due to hand to mouth behavior [34]. Aksomaitiene et al [7] hypothesize that wild bird *C*. *jejuni* strains might be vector for potential transfer to humans especially with overlapping antibiotic resistance patterns with human isolates. Our finding supports the mentioned potential mode of transmission as two ST-2209 isolated from Lithuanian children [35] were assigned to wild bird source with very high posterior probability (~94%).

## Conclusions

This is the first study, where population genetic analyses were conducted to attribute clinical Baltic *C*. *jejuni* isolates to their most likely sources. So far *Campylobacter* studies in Estonia have been mainly focused to poultry, especially broiler chicken meat, as the known main source of *Campylobacter* infections in many countries. The study revealed that clinical cases of *C*. *jejuni* infections in Baltic countries are additionally linked to cattle and wild bird sources. In terms of *Campylobacter* source attribution, there is need to extend surveillance to include also other possible sources e.g. pigs, sheep, pet animals, which currently are not included neither occurrence and molecular epidemiological studies in Estonia, but also not intensively covered in Latvia and Lithuania. To decrease the food derived human infection burden, surveillance of *Campylobacter* contamination within the entire food production and consumption chain is needed, also co-operation with neighboring countries, especially with whom the intensive food trade is present.

## Supporting information

S1 File General_information. General information regarding isolates used in this study.
(TAB)

S2 File GenAlEx parameters. Information regarding parameters used in GenAlEx AMOVA analysis.
(TAB)

S3 File GenAlEx input. Input file with allele data for GenAlEx AMOVA analysis.
(TAB)

S4 File PHYLOViZ parameters. Information regarding parameters used in PHYLOViZ analysis.
(TAB)

S5 File PHYLOViZ first input. Input file with isolate data for PHYLOViZ goeBURST Full MST analysis.
(TAB)

S6 File PHYLOViZ second input. Input file with typing data for PHYLOViZ goeBURST Full MST analysis.
(TAB)

S7 File iSource parameters and random isolates selection. Information regarding parameters used in iSource analysis and Excel formula for random isolate selection for self-attribution analysis used in this study.
(TAB)

S8 File iSource attribution input. Input file with isolate data for iSource attribution analysis.
(TAB)

S9 File iSource self-attribution input. The first input file with isolate data for iSource self-attribution analysis.
(TAB)

S10 File iSource self-attribution input. The second input file with isolate data for iSource self-attribution analysis.
(TAB)

S11 File iSource self-attribution input. The third input file with isolate data for iSource self-attribution analysis.
(TAB)

S12 File iSource Rscript. Modified Rscript used in this study. Original is provided with the iSource software.
(R) Click here for additional data file.
